# Depressed systemic arterial compliance and impaired left ventricular midwall performance in aortic stenosis with concomitant type 2 diabetes: a retrospective cross-sectional study

**DOI:** 10.1186/s12933-019-0894-1

**Published:** 2019-07-17

**Authors:** Ewa Czestkowska, Agnieszka Rożanowska, Dorota Długosz, Klaudiusz Bolt, Jolanta Świerszcz, Olga Kruszelnicka, Bernadeta Chyrchel, Andrzej Surdacki

**Affiliations:** 1 Students’ Scientific Group at the Second Department of Cardiology, Jagiellonian University Medical College, Cracow, Poland; 2 Department of Medical Education, Jagiellonian University Medical College, Cracow, Poland; 3 Department of Coronary Artery Disease and Heart Failure, Jagiellonian University Medical College, Cracow, Poland; 4 Second Department of Cardiology, Jagiellonian University Medical College, 17 Kopernika Street, PL31-501 Cracow, Poland

**Keywords:** Aortic stenosis, Arterial compliance, Left ventricular systolic function, Type 2 diabetes mellitus

## Abstract

**Background:**

Degenerative aortic stenosis (AS), a disease of the elderly, frequently coexists with concomitant diseases, including type 2 diabetes (T2DM) which amplifies the cardiovascular (CV) risk. T2DM affects left ventricular (LV) structure and function via hemodynamic and metabolic factors. In concentric LV geometry, typical for AS, indices of LV midwall mechanics are better estimates of LV function than ejection fraction (EF). Effects of T2DM coexisting with AS on circumferential LV midwall systolic function and large artery properties have not been reported so far. Our aim was to compare characteristics of AS patients with and without T2DM, with a focus on LV midwall systolic function and arterial compliance.

**Methods:**

Medical records of 130 electively hospitalized patients with moderate or severe isolated degenerative AS were retrospectively analyzed. Exclusion criteria included clinical instability, atrial fibrillation, coronary artery disease and relevant non-cardiac diseases. From in-hospital echocardiography and blood pressure, we calculated LV midwall fractional shortening (mwFS), circumferential end-systolic LV wall stress (cESS) and valvulo-arterial impedance (Zva), estimates of LV afterload, as well as systemic arterial compliance.

**Results:**

Patients with (n = 50) and without T2DM (n = 80) did not differ in age, AS severity, LV mass and LV diastolic diameter. T2DM patients exhibited elevated cESS (247 ± 105 vs. 209 ± 84 hPa, p = 0.025) and Zva (5.8 ± 2.2 vs. 5.1 ± 1.8 mmHg per mL/m^2^, p = 0.04), and lower stroke volume index (33 ± 10 vs. 38 ± 12 mL/m^2^, p = 0.01) and systemic arterial compliance (0.53 ± 0.16 vs. 0.62 ± 0.22 mL/m^2^ per mmHg, p = 0.01). mwFS (11.9 ± 3.9 vs. 14.1 ± 3.7%, p = 0.001), but not EF (51 ± 14 vs. 54 ± 13%, p = n.s.), was reduced in T2DM. mwFS and cESS were inversely interrelated in patients both with (r = − 0.59, p < 0.001) and without T2DM (r = − 0.53, p < 0.001) By multiple regression, higher cESS (p < 0.001) and T2DM (p = 0.02) were independent predictors of depressed mwFS.

**Conclusions:**

In AS, coexistent T2DM appears associated with reduced systemic arterial compliance and LV dysfunction at the midwall level, corresponding to slightly depressed myocardial contractility.

**Electronic supplementary material:**

The online version of this article (10.1186/s12933-019-0894-1) contains supplementary material, which is available to authorized users.

## Background

Degenerative aortic stenosis (AS), a disease of the elderly, frequently coexists with concomitant diseases including type 2 diabetes (T2DM) which amplifies the risk of adverse cardiovascular (CV) events in asymptomatic patients [1] and after surgical aortic valve replacement [2]. Regardless of associations with coronary artery disease (CAD) and hypertension, T2DM per se is a risk factor for heart failure (HF) [3–6], influencing left ventricular (LV) structure and function via multiple pathways, including large artery stiffening and direct effects on the myocardium with consequent enhanced LV hypertrophy and LV dysfunction [7–11].

Although a restrictive, not dilated, phenotype of diabetic cardiomyopathy predominates in T2DM without CV disease [10, 12, 13], slight impairment of LV systolic function despite normal ejection fraction (EF) appears also frequent, with combined systolic and diastolic dysfunction in 10–25% of T2DM patients without overt cardiac disease [14–16]. Of note, subclinical circumferential and/or longitudinal LV systolic dysfunction was reported in over one-half of T2DM patients free of CV disease [16]. Additionally, impaired stress-corrected LV midwall fractional shortening (mwFS), detected in almost 40% of T2DM subjects without CV disease, independently predicted CV mortality [17]. Moreover, depressed mwFS predisposed to adverse ischemic and aortic valve-related CV events in asymptomatic AS with a preserved EF [18].

To the best of our knowledge, effects of T2DM coexisting with AS on circumferential LV systolic function at the midwall level, a better estimate of LV function than EF at concentric LV geometry, typical for AS, have not been reported so far. As compared to non-diabetic patients with severe AS, in diabetic AS subjects Lindman et al. [19] found reduced conventional EF (i.e., at the endocardial level) and depressed longitudinal LV systolic function by strain-rate imaging using the speckle tracking method, a trend toward impaired LV diastolic function by tissue Doppler, and similar systemic arterial compliance. On the other hand, Falcão-Pires et al. [20] described significantly lower LV end-diastolic distensibility, enhanced interstitial myocardial fibrosis and reduced cardiomyocyte passive stiffness in AS patients with versus without T2DM, undergoing perioperative LV biopsies, however the subgroups had similar EF. Our aim was to compare characteristics of AS patients with and without T2DM, with a focus on LV systolic function at the midwall level and systemic arterial compliance.

## Methods

### Patients

We retrospectively analyzed medical records of clinically stable patients hospitalized on an elective basis in our center during 2013–2018 with a final diagnosis of isolated moderate or severe degenerative AS-defined as aortic valve area ≤ 1.5 cm^2^ (by the continuity equation), supported by additional measures (mean aortic pressure gradient, aortic valve area index and maximal aortic jet velocity) in case of any doubts with regard to AS severity, i.e. in accordance with an integrative approach to grading AS [21, 22]. Exclusion criteria included: more than mild aortic regurgitation or disease of another valve, atrial fibrillation, a history of myocardial infarction or coronary revascularization, diameter narrowings of ≥ 50% in major epicardial artery segments on coronary angiography, estimated glomerular filtration rate < 30 mL/min per 1.73 m^2^ (by the CKD-EPI formula), other relevant non-cardiac coexistent diseases except for T2DM and well-controlled hypertension, and the use of sodium-glucose co-transporter 2 (SGLT-2) inhibitors or glucagon-like peptide 1 receptor agonists.

On the basis of the exclusion criteria, out of 335 pre-screened subjects, 130 AS patients in sinus rhythm with an adequate echocardiographic image quality and complete data (50 with previously diagnosed T2DM and 80 without diabetes) entered the final analysis.

### Data extraction and additional calculations

In-hospital echocardiography was performed on an ultrasound device (Vivid 8; GE Healthcare, Chicago, IL, USA) by a recognized sonographer. From routine in-hospital echocardiographic records we extracted EF, calculated from recorded 2D-images by means of the modified Simpson’s rule and validated by one of the senior authors, while LV mass was derived by the Devereux formula, in accordance with the current practice guidelines [23]. Additionally, from echocardiography and mean in-hospital blood pressure (computed from all in-hospital blood pressure measurements), we calculated valvulo-arterial impedance (Zva)—an index of the sum of valvular and arterial components of LV afterload, and systemic arterial compliance, as previously proposed [24]. Zva was derived from systolic blood pressure, mean aortic pressure gradient and stroke volume index, whereas systemic arterial compliance from stroke volume index and pulse pressure [24], as in our earlier reports [25, 26].

Also, in agreement with a simplified cylindrical LV model [27–29], from 2D-guided M-mode LV measurements and blood pressure, mwFS and circumferential end-systolic LV midwall stress (cESS) at the LV minor axis, an estimate of afterload at the ventricular level, were computed for the following reasons. First, vectors of LV midwall stress and fiber shortening are oriented in the same direction because circumferential fibers predominate at the LV midwall equator, in contrast to the subendocardial layer where longitudinal fibers are more prevalent [28]. Second, as mwFS was derived on the basis of a constant volume of the myocardial “shell” between the midwall and the endocardium [27], the proposed approach takes into consideration systolic migration of midwall fibers from the middle line towards epicardium, providing a better index of LV performance than EF which overestimates LV function at concentric LV geometry [28].

As previously proposed [27], cESS and mwFS were calculated according to the following formulas:

$$ \begin{aligned} {\text{cESS }}\left[ {\text{hPa}} \right] \, =  1.333 { } \times \, \left( {{\text{SBP}} + {\text{PG}}_{ \hbox{max} } } \right) \, \times \, \left( {{\text{LVs}}/ 2} \right)^{ 2} \hfill \\ \times {{\left[ { 1 { } + \left\{ {{{\left[ {\left( {{\text{LVs}}/ 2} \right) + {\text{PWs}}} \right]^{2} } \mathord{\left/ {\vphantom {{\left[ {\left( {{\text{LVs}}/ 2} \right) + {\text{PWs}}} \right]^{2} } {\left[ {\left( {{\text{LVs}}/ 2} \right) + {\text{PWs/2}}} \right]^{2} }}} \right. \kern-0pt} {\left[ {\left( {{\text{LVs}}/ 2} \right) + ({\text{PWs/2}})} \right]^{2} }}} \right\}} \right]} \mathord{\left/ {\vphantom {{\left[ { 1 { } + \left\{ {{{\left[ {\left( {{\text{LVs}}/ 2} \right) + {\text{PWs}}} \right]^{2} } \mathord{\left/ {\vphantom {{\left[ {\left( {{\text{LVs}}/ 2} \right) + {\text{PWs}}} \right]^{2} } {\left[ {\left( {{\text{LVs}}/ 2} \right) + {\text{PWs/2}}} \right]^{2} }}} \right. \kern-0pt} {\left[ {\left( {{\text{LVs}}/ 2} \right) + {\text{PWs/2}}} \right]^{2} }}} \right\}} \right]} {\left\{ {\left[ {\left[ {\left( {{\text{LVs}}/ 2} \right) + {\text{PWs}}} \right]^{2} - \left( {{{\text{LVs}} \mathord{\left/ {\vphantom {{\text{LVs}} 2}} \right. \kern-0pt} 2}} \right)^{2} } \right]} \right\}}}} \right. \kern-0pt} {\left\{ {\left[ {\left[ {\left( {{\text{LVs}}/ 2} \right) + {\text{PWs}}} \right]^{2} - \left( {{{\text{LVs}} \mathord{\left/ {\vphantom {{\text{LVs}} 2}} \right. \kern-0pt} 2}} \right)^{2} } \right]} \right\}}}, \hfill \\ \end{aligned} $$

$$ {\text{mwFS }} = \, \left\{ {{{\left[ {\left( {{\text{LVd }} + {\text{ PWd}}/ 2 { } + {\text{ IVSd}}/ 2} \right) \, {-} \, \left( {{\text{LVs }} + {\text{ Hs}}/ 2} \right)} \right]} \mathord{\left/ {\vphantom {{\left[ {\left( {{\text{LVd }} + {\text{ PWd}}/ 2 { } + {\text{ IVSd}}/ 2} \right) \, {-} \, \left( {{\text{LVs }} + {\text{ Hs}}/ 2} \right)} \right]} {\left( {{\text{LVd }} + {\text{ PWd}}/ 2 { } + {\text{ IVSd}}/ 2} \right)}}} \right. \kern-0pt} {\left( {{\text{LVd }} + {\text{ PWd}}/ 2 { } + {\text{ IVSd}}/ 2} \right)}}} \right\} \, \times { 1}00\% , $$

where SBP is systolic blood pressure [mmHg], PG_max_: maximal transvalvular aortic pressure gradient [mmHg], LVd: LV end-diastolic diameter [cm]; LVs: LV end-systolic diameter [cm]; PWd: LV posterior wall thickness at end-diastole; PWs: LV posterior wall thickness at end-systole [cm]; IVSd: interventricular septum thickness at end-diastole [cm]; Hs/2: systolic LV inner shell myocardial thickness, computed in agreement with the assumption of a constant volume of the LV inner myocardial shell throughout the cardiac cycle [27], and 1.333 is a conversion factor from mmHg into hPa.

### Statistical analysis

Patients’ characteristics were compared between subjects with and without T2DM by a 2-sided Student’s t test (or a Welch’s test for inhomogeneous variance) and the Chi square test for continuous and categorical data, respectively. The homogeneity of variance was verified by Levene’s test. Bivariate correlations were estimated by Pearson’s correlation coefficients (r). Multiple linear regression was used to identify independent determinants of mwFS. Additionally, assuming low mwFS as mwFS < 14% in women and < 16% in men, we performed stepwise logistic regression with low mwFS as a dichotomous dependent variable, and the following potential predictors: cESS, systolic blood pressure, hypertension and metabolic syndrome, separately for diabetic and non-diabetic AS patients. A p-value below 0.05 was assumed significant.

## Results

### Comparison of clinical and hemodynamic characteristics in aortic stenosis patients with and without concomitant T2DM

Patients with and without T2DM did not differ in clinical characteristics except for higher body-mass index in T2DM (Table 1).

**Table 1:** Clinical characteristics in patients with and without concomitant T2DM

Characteristic	T2DM n = 50	No diabetes n = 80	p value
Age (years)	70 ± 9	70 ± 11	n.s.
Men/Women, n (%)	23/27 (46/54%)	44/36 (55/45%)	n.s.
Symptoms, n (%)^a^	31 (62%)	41 (51%)	n.s.
Hypertension, n (%)	45 (90%)	62 (78%)	n.s.
Chronic kidney disease, n (%)	16 (32%)	17 (21%)	n.s.
Body-mass index (kg/m^2^)	31 ± 5	28 ± 4	0.003
Mean blood pressure (mm Hg)	90 ± 10	89 ± 10	n.s.
Systolic blood pressure (mm Hg)	131 ± 16	130 ± 18	n.s.
Glycated hemoglobin (%)	7.1 ± 0.8	–	–
Medications, n (%)			
ACEI or ARB	30 (60%)	43 (54%)	n.s.
Beta-blocker	25 (50%)	34 (43%)	n.s.
Diuretic	20 (40%)	27 (34%)	n.s.
Calcium channel blockers	17 (34%)	24 (30%)	n.s.
Statin	38 (76%)	50 (62%)	n.s.
Metformin	47 (94%)	–	–
Insulin	16 (32%)	–	–

Data are shown as mean ± SD or n (%)

*ACEI* angiotensin-converting enzyme inhibitors, *ARB* angiotensin receptor blockers

^a^A history of heart failure, syncope or angina linked to AS

**Fig. 1:**
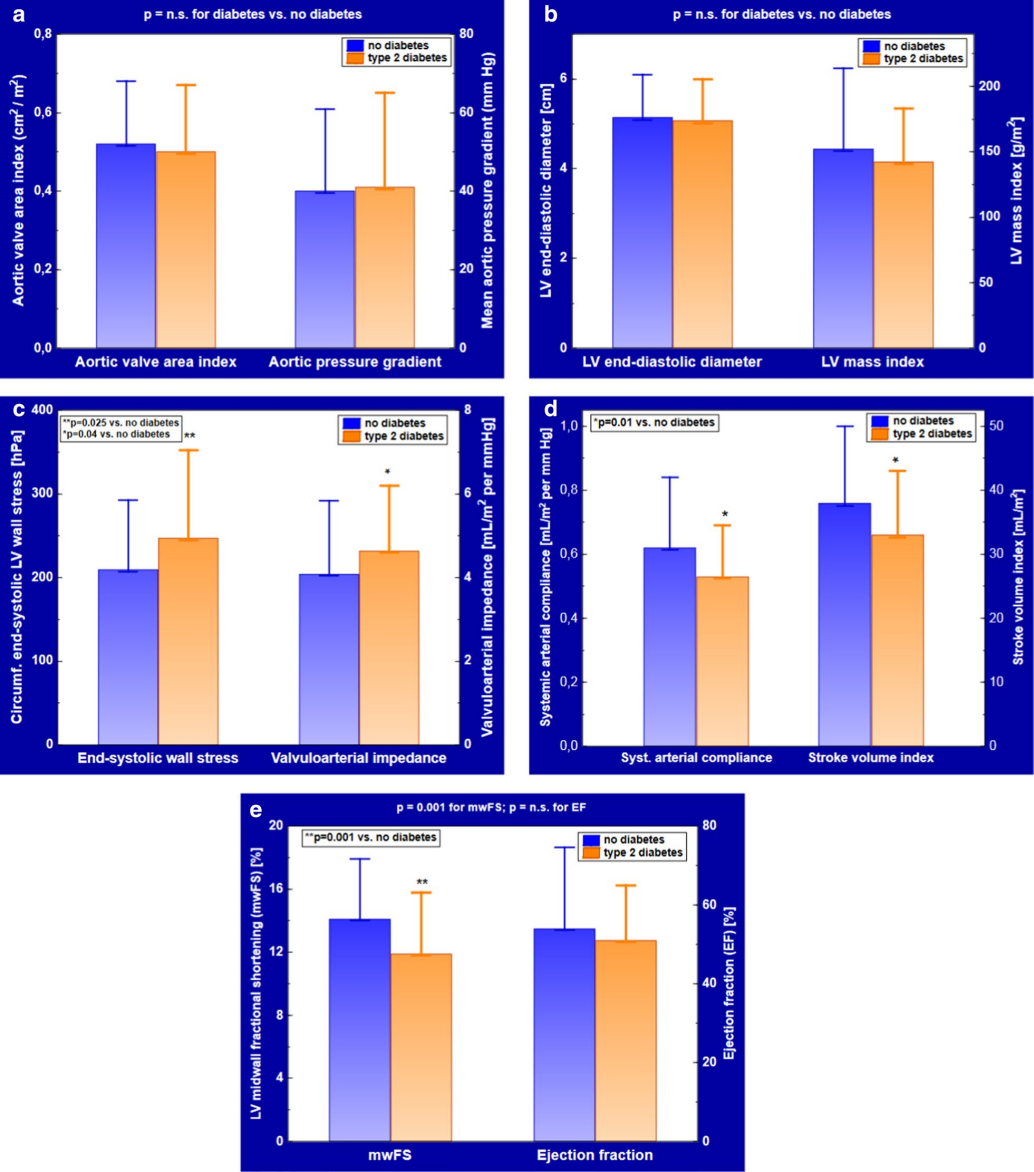
Hemodynamic characteristics by T2DM status. **a** Aortic valve area index and mean aortic pressure gradient; **b** LV end-diastolic diameter, a raw estimate of LV preload, and LV mass index; **c** circumferential end-systolic LV midwall stress and valvulo-arterial impedance, indices of LV afterload; **d** systemic arterial compliance and LV stroke volume index; **e** LV midwall fractional shortening and ejection fraction. Data are shown as mean ± SD

Patients’ hemodynamic characteristics by T2DM status are shown in Table 2. The severity of AS, estimated by aortic valve area index and mean aortic pressure gradient, was comparable in patients with and without concomitant T2DM (Fig. 1a). There were also no intergroup differences in LV end-diastolic diameter and LV mass index (Fig. 1b). Both measures of LV afterload, cESS and Zva were elevated in T2DM (Fig. 1c), whereas systemic arterial compliance was depressed, due to a lower stroke volume index (Fig. 1d) at a similar pulse pressure. Of note, mwFS—but not EF—was significantly reduced in T2DM (Fig. 1e).

**Table 2:** Hemodynamic characteristics of AS patients with and without concomitant T2DM

Characteristic	T2DM n = 50	No diabetes n = 80	p value
Aortic valve area index (cm^2^/m^2^)	0.50 ± 0.17	0.52 ± 0.16	n.s.
Mean aortic pressure gradient (mm Hg)	41 ± 24	40 ± 21	n.s.
LV end-diastolic diameter (cm)	5.1 ± 0.9	5.1 ± 1.0	n.s.
LV mass index (g/m^2^)	143 ± 41	152 ± 62	n.s.
LV ejection fraction (%)	51 ± 14	54 ± 13	n.s.
LV midwall fractional shortening (%)	11.9 ± 3.9	14.1 ± 3.7	0.001
Low LV midwall fractional shortening, n (%)	39 (78%)	48 (60%)	0.03
Stroke volume index (mL/m^2^)	33 ± 10	38 ± 12	0.01
cESS (hPa)	247 ± 105	209 ± 84	0.025
Zva (mm Hg per mL/m^2^)	5.8 ± 2.2	5.1 ± 1.8	0.04
Pulse pressure (mm Hg)	63 ± 14	64 ± 17	n.s.
Systemic arterial compliance (mL/m^2^ per mm Hg)	0.53 ± 0.16	0.62 ± 0.22	0.01

Data are shown as mean ± SD

*cESS* circumferential end-systolic LV midwall stress, *LV* left ventricular, *Zva* valvulo-arterial impedance

### Comparison of the relationship between mwFS and cESS in aortic stenosis patients with and without concomitant T2DM

**Fig. 2:**
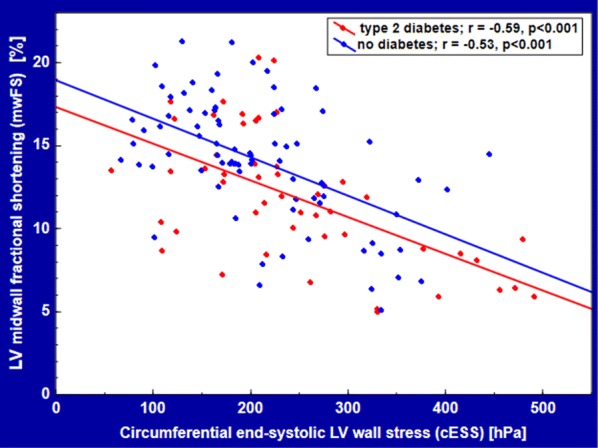
LV midwall fractional shortening plotted against circumferential end-systolic LV midwall stress in AS patients with and without T2DM

mwFS and cESS were negatively related with each other in patients with T2DM (r = − 0.59, p < 0.001) and without T2DM (r = − 0.53, p < 0.001) (Fig. 2). mwFS, plotted against cESS, was lower in AS with T2DM compared to AS without T2DM for each given value of cESS (Fig. 2). This effect was confirmed by multiple regression: mwFS was depressed in T2DM subjects (mean β ± SEM: −0.17 ± 0.07, p = 0.02), independently of a strong inverse association between mwFS and cESS (β = − 0.54 ± 0.07, p < 0.001).

As a continuous variable, mwFS was unrelated to any other patients’ characteristics than cESS, including also systemic arterial compliance or symptomatic status in either diabetic AS subjects, their non-diabetic counterparts or the whole study group (p > 0.15).

By stepwise logistic regression, exclusively increased cESS independently predicted low mwFS, when analyzing diabetic and non-diabetic AS patients separately (mean odds ratio [OR] per 10-hPa increment: 1.11 [confidence interval: 1.01–1.23], p = 0.035 for subjects with T2DM; OR = 1.13 [1.05–1.21], p = 0.001 for subjects without diabetes).

## Discussion

Our salient finding was reduced systemic arterial compliance and lower mwFS, reflecting LV systolic performance at the midwall level, which was not entirely explained by higher cESS, an index of LV afterload, in patients with moderate or severe AS and concomitant T2DM.

### Comparison with other studies

The prevalence of low mwFS was 78% in our AS patients with concomitant T2DM, i.e. higher than the respective proportion (34–52%) of subjects with impaired LV midwall systolic function among T2DM patients without overt CV disease, participating in the DYDA [15] and SHOCKWAVE [16] studies. However, none of those T2DM subjects exhibited significant valvular heart disease [15, 16], whereas our patients had AS and T2DM, both of which predispose to LV systolic dysfunction and have been referred to as “an ominous combination” by Banovic et al. [30]. The proportion of impaired circumferential LV systolic function at the midwall level was 24%, 28% and 30% in normal weight, overweight and obese non-diabetic patients with mild or moderate asymptomatic isolated AS in the SEAS cohort [31], and 71% in asymptomatic severe AS, whose vast majority (94%) was free of diabetes [1]. Because our AS subjects had moderate or severe AS and were mainly overweight or obese, the high prevalence (60%) of low mwFS even in our AS patients without diabetes is generally consistent with those reports [1, 31].

Notably, diabetic subjects were excluded from the SEAS [31] and ASTRONOMER [32] studies, which investigated determinants of longitudinal [32] and circumferential midwall [31] LV systolic function in large groups of patients with mild or moderate asymptomatic isolated AS. Moreover, Lindman et al. [19], who reported longitudinal LV systolic dysfunction by strain-rate imaging by means of the speckle tracing method in severe AS with versus without concomitant diabetes, did not analyze circumferential midwall LV function, and did not adjust peak longitudinal systolic strain for LV afterload. Therefore, our study is first to demonstrate associations of T2DM with subclinical circumferential LV systolic dysfunction at the midwall level AS, irrespective of higher cESS. Accordingly, this finding supplements earlier evidence from the Multi-Ethnic Study of Atherosclerosis (MESA) which detected an association of diabetes with regional LV systolic dysfunction by circumferential strain imaging in four LV midwall segments using cardiac magnetic resonance in subjects without clinically recognized CV disease [33].

Keeping in mind numerous reports of elevated arterial stiffness and reduced vascular compliance in T2DM [11], lower systemic arterial compliance in our diabetic AS subjects does not appear unexpected, although Lindman et al. [19] did not observed a significant difference in this parameter according to diabetes status in severe AS.

### Mechanistic considerations

Our preliminary observations, based on a retrospective cross-sectional data analysis, allows only cautious interpretations in term of possible mechanisms. Nevertheless, as metabolic factors have been implicated in the pathogenesis of both diabetic cardiomyopathy and altered vascular properties, multiple detrimental pathways are likely to contribute to CV damage also in AS [9–11, 30, 34, 35]. That, in contrast to previously reported associations of poor glycemic control with lower ascendic aortic distensibility [36] and higher aortic pulse wave velocity in T2DM [37], systemic arterial compliance was unrelated to glycated hemoglobin in our diabetic AS subjects, might be due to multifactorial nature of vascular abnormalities in T2DM, including not only deposition of advanced glycation end-products, but also insulin resistance, impaired nitric oxide bioavailability, oxidative stress, enhanced fibrosis and inflammatory activation [11, 30]. Likewise, a variety of mechanisms underlying CV damage in T2DM [9–11, 30, 34, 35, 38] could be accountable for no relations between symptomatic status, systemic arterial compliance and LV function in the present study.

Additionally, in the SEAS [31] and ASTRONOMER [32] studies, subclinical circumferential [31] and longitudinal [32] LV systolic dysfunction was reported already in non-diabetic AS patients with concomitant metabolic syndrome [32] or obesity [31], well-known predecessors of T2DM, and metabolic syndrome was associated with marginally lower systemic arterial compliance in the ASTRONOMER cohort [32], which points into the relevance of insulin resistance/hyperinsulinemia, accompanied by altered myocardial substrate metabolism (a shift from glucose to fatty acids oxidation) and chronic low-grade inflammatory activation [10, 34, 35, 38], for cardiac and vascular damage before the onset of T2DM. This concept is also consistent with a lower stress-corrected mwFS in adolescents and young adults with impaired fasting glucose, i.e. prediabetic state, compared to those with normal glucose tolerance in the Strong Heart Study [39]. Moreover, insulin resistance was identified as an independent correlate of reduced large artery compliance in asymptomatic young adults participating in the Bogalusa Heart Study [40]. Accordingly, that we observed the coexistence of reduced mwFS and systemic arterial compliance in our AS patients with versus without T2DM, might indicate a highly adverse constellation [30] (probably present for many years before T2DM onset) accountable for consequent depressed stroke volume index, also found in our diabetic AS subjects. Notably, as systolic blood pressure, mean aortic gradient and pulse pressure were similar according to T2DM status, reduced stroke volume index was largely responsible for increased Zva and decreased systemic arterial compliance in our AS patients with concomitant T2DM. Importantly, low stroke volume index, an independent predictor of incident HF in the Strong Heart Study population [41], is also a hallmark of increased risk of mortality in severe AS with preserved EF [42] and after transcatheter aortic valve implantation [43].

### Aortic stenosis with concomitant T2DM – a clinical challenge

Patients with both AS and T2DM are a specific group because renin-angiotensin system inhibitors, a powerful tool for cardioprotection and nephroprotection in T2DM, are perceived as relatively contraindicated in severe AS, due to the putative risk of hypotension. Therefore, alternative strategies to improve CV outcome are necessary in patients with combined AS and T2DM, in whom the risk of adverse CV events is elevated by 4- to sixfold in asymptomatic severe AS [1], renin-angiotensin antagonists are frequently underused, while surgical or transcatheter aortic valve implantation are limited to subjects with recognized indications to these procedures. Keeping in mind the potential relevance of metabolic factors for CV damage in AS with coexistent T2DM [30], optimized long-term glycemic control and lifestyle interventions in T2DM and prediabetes might modulate a corollary of detrimental pathways which contribute to accelerated development of HF in patients with combined AS and T2DM.

Irrespective of the degree of glycemic control, novel antidiabetic drugs such as SGLT-2 inhibitors—recommended for patients with T2DM and established CV disease, especially HF [44]—can have a potential to improve prognosis also in AS. Hypothetically, the ability of SGLT-2 inhibitors to prevent both development of HF and HF hospitalizations in patients with prevalent HF [44], linked to multiple mechanisms, including reduced LV loading conditions and decreased arterial stiffness [35, 44–48], could translate into delayed onset of symptoms in AS patients on medical therapy. Nevertheless, CV effects of SGLT-2 antagonists in T2DM with concomitant AS still remain to be investigated.

Finally, reduced mwFS or depressed systemic arterial compliance might possibly be helpful in risk stratification of asymptomatic AS subjects with concomitant T2DM. In fact, subclinical circumferential LV midwall dysfunction was independently related to incident HF over a 5-year follow-up in the MESA participants free of known CV disease, irrespective of diabetes status and end-systolic LV wall stress [33]. In addition, aortic pulse wave velocity was independently related to pre- and post-operative NYHA class in AS subjects undergoing surgical valve replacement [49]. Nevertheless, large-scale prospective studies are warranted to assess potential clinical utility of measures of arterial stiffness or compliance, as well as novel indices of LV function, such as mwFS or recently proposed diastolic wall strain [50] in asymptomatic AS patients.

### Study limitations

First, our findings have been based on a single-center retrospective analysis of medical records of patients hospitalized for AS, in whom the diagnosis of T2DM had been established prior to admission. Nevertheless, T2DM diagnosis has been verified in any dubious cases. Additionally, in order to increase the homogeneity of the study population, we had excluded subjects with relevant non-cardiac disorders and underlying cardiac pathology other than pure AS, especially without CAD. Second, novel echocardiographic techniques, such as tissue Doppler or strain imaging, are appropriate for the quantification of subclinical LV dysfunction, however, we were only able to compute mwFS from available routine echocardiographic records, and exclusively good-quality images entered the final analysis. Third, systemic arterial compliance was estimated by a simple index derived from stroke volume index and pulse pressure, whereas a complex approach, including arterial tonometry, pressure-flow analysis and 24-h blood pressure monitoring, is more suitable to investigate arterial properties, all the more because Chirinos et al. [51] have recently demonstrated the association of T2DM with arterial stiffening and abnormal pulsatile arterial hemodynamics in HF with preserved EF. Fourth, medical therapy was not uniform in the study group. Nonetheless, the proportion of renin-angiotensin system antagonists and beta-blockers was similar in patients with and without T2DM, almost all T2DM subjects were on metformin and none of them was receiving SGLT-2 inhibitors or glucagon-like peptide 1 receptor agonists. Finally, owing to limited availability of longitudinal medical records, we were unable to investigate either the previously suggested effect of T2DM on the progression of AS severity [30] or AS-related outcomes.

## Conclusions

In AS, coexistent T2DM appears associated with reduced systemic arterial compliance and impaired LV systolic function at the midwall level, corresponding to slightly depressed myocardial contractility. Further studies are warranted to investigate possible strategies which might improve LV performance and delay onset of symptoms in AS with concomitant T2DM.

## Additional file

**Additional file 1.** “T2DM” (0 = no diabetes; 1 = T2DM); “cESS” (circumferential end-systolic LV midwall stress) [hPa]; “mwFS” (LV midwall fractional shortening) [%]; “SAC” (systemic arterial compliance)” [mL/m^2^ per mm Hg].

## Data Availability

The key data that support the findings of this study are included in the published article and its Additional file 1.

## Abbreviations

ACEI: angiotensin-converting enzyme inhibitors
ARB: angiotensin receptor blockers
AS: aortic stenosis
CAD: coronary artery disease
CV: cardiovascular
cESS: circumferential end-systolic LV midwall stress
T2DM: type 2 diabetes mellitus
EF: ejection fraction
HF: heart failure
LV: left ventricular
mwFS: LV midwall fractional shortening
OR: odds ratio
SD: standard deviation
SEM: standard error of the mean
SGLT-2: sodium-glucose co-transporter 2
Zva: valvulo-arterial impedance

## References

[1] Cioffi G, Faggiano P, Vizzardi E, Tarantini L, Cramariuc D, Gerdts E, de Simone G (2011). Prognostic effect of inappropriately high left ventricular mass in asymptomatic severe aortic stenosis. Heart.

[2] Ram E, Kogan A, Levin S, Fisman EZ, Tenenbaum A, Raanani E, Sternik L (2019). Type 2 diabetes mellitus increases long-term mortality risk after isolated surgical aortic valve replacement. Cardiovasc Diabetol..

[3] Kannel WB, McGee DL (1979). Diabetes and cardiovascular disease. The Framingham study. JAMA..

[4] Aronow WS, Ahn C (1999). Incidence of heart failure in 2,737 older persons with and without diabetes mellitus. Chest.

[5] Gottdiener JS, Arnold AM, Aurigemma GP, Polak JF, Tracy RP, Kitzman DW, Gardin JM, Rutledge JE, Boineau RC (2000). Predictors of congestive heart failure in the elderly: the Cardiovascular Health Study. J Am Coll Cardiol.

[6] De Simone G, Devereux RB, Chinali M, Lee ET, Galloway JM, Barac A, Panza JA, Howard BV (2010). Diabetes and incident heart failure in hypertensive and normotensive participants of the Strong Heart Study. J Hypertens.

[7] Bella JN, Devereux RB, Roman MJ, Palmieri V, Liu JE, Paranicas M, Welty TK, Lee ET, Fabsitz RR, Howard BV (2001). Separate and joint effects of systemic hypertension and diabetes mellitus on left ventricular structure and function in American Indians (the Strong Heart Study). Am J Cardiol.

[8] Palmieri V, Bella JN, Arnett DK, Liu JE, Oberman A, Schuck MY, Kitzman DW, Hopkins PN, Morgan D, Rao DC, Devereux RB (2001). Effect of type 2 diabetes mellitus on left ventricular geometry and systolic function in hypertensive subjects: hypertension Genetic Epidemiology Network (HyperGEN) study. Circulation.

[9] Ernande L, Derumeaux G (2012). Diabetic cardiomyopathy: myth or reality?. Arch Cardiovasc Dis..

[10] Seferović PM, Paulus WJ (2015). Clinical diabetic cardiomyopathy: a two-faced disease with restrictive and dilated phenotypes. Eur Heart J.

[11] Prenner SB, Chirinos JA (2015). Arterial stiffness in diabetes mellitus. Atherosclerosis..

[12] Liu JE, Palmieri V, Roman MJ, Bella JN, Fabsitz R, Howard BV, Welty TK, Lee ET, Devereux RB (2001). The impact of diabetes on left ventricular filling pattern in normotensive and hypertensive adults: the Strong Heart Study. J Am Coll Cardiol.

[13] Kiencke S, Handschin R, von Dahlen R, Muser J, Brunner-Larocca HP, Schumann J, Felix B, Berneis K, Rickenbacher P (2010). Pre-clinical diabetic cardiomyopathy: prevalence, screening, and outcome. Eur J Heart Fail.

[14] Ernande L, Bergerot C, Rietzschel ER, De Buyzere ML, Thibault H, Pignonblanc PG, Croisille P, Ovize M, Groisne L, Moulin P, Gillebert TC, Derumeaux G (2011). Diastolic dysfunction in patients with type 2 diabetes mellitus: is it really the first marker of diabetic cardiomyopathy?. J Am Soc Echocardiogr.

[15] Giorda CB, Cioffi G, de Simone G, Di Lenarda A, Faggiano P, Latini R, Lucci D, Maggioni AP, Tarantini L, Velussi M, Verdecchia P, Comaschi M, Investigators D (2011). Predictors of early-stage left ventricular dysfunction in type 2 diabetes: results of DYDA study. Eur J Cardiovasc Prev Rehabil..

[16] Faden G, Faganello G, De Feo S, Berlinghieri N, Tarantini L, Di Lenarda A, Faggiano P, Cioffi G (2013). The increasing detection of asymptomatic left ventricular dysfunction in patients with type 2 diabetes mellitus without overt cardiac disease: data from the SHORTWAVE study. Diabetes Res Clin Pract.

[17] Cioffi G, Rossi A, Targher G, Zoppini G, de Simone G, Devereux RB, Bonora E, Vassanelli C (2014). Usefulness of subclinical left ventricular midwall dysfunction to predict cardiovascular mortality in patients with type 2 diabetes mellitus. Am J Cardiol..

[18] Cioffi G, Mazzone C, Barbati G, Rossi A, Nistri S, Ognibeni F, Tarantini L, Di Lenarda A, Faggiano P, Pulignano G, Stefenelli C, de Simone G (2016). Value of combined circumferential and longitudinal left ventricular systolic dysfunction to predict adverse outcome in patients with asymptomatic aortic stenosis. J Heart Valve Dis.

[19] Lindman BR, Arnold SV, Madrazo JA, Zajarias A, Johnson SN, Pérez JE, Mann DL (2011). The adverse impact of diabetes mellitus on left ventricular remodeling and function in patients with severe aortic stenosis. Circ Heart Fail..

[20] Falcão-Pires I, Hamdani N, Borbély A, Gavina C, Schalkwijk CG, van der Velden J, van Heerebeek L, Stienen GJ, Niessen HW, Leite-Moreira AF, Paulus WJ (2011). Diabetes mellitus worsens diastolic left ventricular dysfunction in aortic stenosis through altered myocardial structure and cardiomyocyte stiffness. Circulation.

[21] Baumgartner H, Falk V, Bax JJ, Bonis M, Hamm C, Holm PJ, Iung B, Lancellotti P, Lansac E, Munoz DR, Rosenhek R, Sjögren J, Mas PT, Vahanian A, Walther T, Wendler O, Windecker S (2018). Zamorano JL [2017 ESC/EACTS Guidelines for the management of valvular heart disease]. Kardiol Pol.

[22] Baumgartner H, Hung J, Bermejo J, Chambers JB, Edvardsen T, Goldstein S, Lancellotti P, LeFevre M, Miller F, Otto CM (2017). Recommendations on the echocardiographic assessment of aortic valve stenosis: a focused update from the European Association of Cardiovascular Imaging and the American Society of Echocardiography. Eur Heart J Cardiovasc Imaging..

[23] Lang RM, Badano LP, Mor-Avi V, Afilalo J, Armstrong A, Ernande L, Flachskampf FA, Foster E, Goldstein SA, Kuznetsova T, Lancellotti P, Muraru D, Picard MH, Rietzschel ER, Rudski L, Spencer KT, Tsang W, Voigt JU (2015). Recommendations for cardiac chamber quantification by echocardiography in adults: an update from the American Society of Echocardiography and the European Association of Cardiovascular Imaging. Eur Heart J Cardiovasc Imaging..

[24] Briand M, Dumesnil JG, Kadem L, Tongue AG, Rieu R, Garcia D, Pibarot P (2005). Reduced systemic arterial compliance impacts significantly on left ventricular afterload and function in aortic stenosis: implications for diagnosis and treatment. J Am Coll Cardiol.

[25] Kruszelnicka O, Chmiela M, Bobrowska B, Świerszcz J, Bhagavatula S, Bednarek J, Surdacki A, Nessler J, Hryniewiecki T (2015). Depressed systemic arterial compliance is associated with the severity of heart failure symptoms in moderate-to-severe aortic stenosis: a cross-sectional retrospective study. Int J Med Sci..

[26] Długosz D, Bolt K, Sam WS, Nawara T, Kruszelnicka O, Chyrchel B, Surdacki A (2018). Excessive left ventricular hypertrophy in moderate degenerative aortic stenosis: an ineffective compensatory mechanism triggered by primary myocardial dysfunction and enhanced by concomitant mild renal impairment?. Kardiol Pol..

[27] De Simone G, Devereux RB, Roman MJ, Ganau A, Saba PS, Alderman MH, Laragh JH (1994). Assessment of left ventricular function by the midwall fractional shortening/end-systolic stress relation in human hypertension. J Am Coll Cardiol.

[28] Aurigemma GP, Silver KH, Priest MA, Gaasch WH (1995). Geometric changes allow normal ejection fraction despite depressed myocardial shortening in hypertensive left ventricular hypertrophy. J Am Coll Cardiol.

[29] Chyrchel B, Długosz D, Bolt K, Kruszelnicka O, Dziewierz A, Świerszcz J, Wieczorek-Surdacka E, Hryniewiecki T, Surdacki A (2018). Association of inadequately low left ventricular mass with enhanced myocardial contractility in severe degenerative aortic stenosis. J Clin Med..

[30] Banovic M, Athithan L, McCann GP (2019). Aortic stenosis and diabetes mellitus: an ominous combination. Diab Vasc Dis Res..

[31] Lund BP, Gohlke-Bärwolf C, Cramariuc D, Rossebø AB, Rieck AE, Gerdts E (2010). Effect of obesity on left ventricular mass and systolic function in patients with asymptomatic aortic stenosis (a Simvastatin Ezetimibe in Aortic Stenosis [SEAS] substudy). Am J Cardiol.

[32] Pagé A, Dumesnil JG, Clavel MA, Chan KL, Teo KK, Tam JW, Mathieu P, Després JP, Pibarot P, ASTRONOMER Investigators (2010). Metabolic syndrome is associated with more pronounced impairment of left ventricle geometry and function in patients with calcific aortic stenosis: a substudy of the ASTRONOMER (Aortic Stenosis Progression Observation Measuring Effects of Rosuvastatin). J Am Coll Cardiol..

[33] Choi EY, Rosen BD, Fernandes VR, Yan RT, Yoneyama K, Donekal S, Opdahl A, Almeida AL, Wu CO, Gomes AS, Bluemke DA, Lima JA (2013). Prognostic value of myocardial circumferential strain for incident heart failure and cardiovascular events in asymptomatic individuals: the Multi-Ethnic Study of Atherosclerosis. Eur Heart J.

[34] Galderisi M (2006). Diastolic dysfunction and diabetic cardiomyopathy: evaluation by Doppler echocardiography. J Am Coll Cardiol.

[35] Lahnwong S, Chattipakorn SC, Chattipakorn N (2018). Potential mechanisms responsible for cardioprotective effects of sodium-glucose co-transporter 2 inhibitors. Cardiovasc Diabetol..

[36] Swoboda PP, Erhayiem B, Kan R, McDiarmid AK, Garg P, Musa TA, Dobson LE, Witte KK, Kearney MT, Barth JH, Ajjan R, Greenwood JP, Plein S (2018). Cardiovascular magnetic resonance measures of aortic stiffness in asymptomatic patients with type 2 diabetes: association with glycaemic control and clinical outcomes. Cardiovasc Diabetol..

[37] Kozakova M, Morizzo C, Fraser AG, Palombo C (2017). Impact of glycemic control on aortic stiffness, left ventricular mass and diastolic longitudinal function in type 2 diabetes mellitus. Cardiovasc Diabetol..

[38] Boudina S, Abel ED (2007). Diabetic cardiomyopathy revisited. Circulation.

[39] De Marco M, de Simone G, Roman MJ, Chinali M, Lee ET, Calhoun D, Howard BV, Devereux RB (2011). Cardiac geometry and function in diabetic or prediabetic adolescents and young adults: the Strong Heart Study. Diabetes Care.

[40] Bhuiyan AR, Srinivasan SR, Chen W, Paul TK, Berenson GS (2006). Correlates of vascular structure and function measures in asymptomatic young adults: the Bogalusa Heart Study. Atherosclerosis..

[41] De Marco M, Gerdts E, Mancusi C, Roman MJ, Lønnebakken MT, Lee ET, Howard BV, Devereux RB, de Simone G (2017). Influence of left ventricular stroke volume on incident heart failure in a population with preserved ejection fraction (from the Strong Heart Study). Am J Cardiol.

[42] Rusinaru D, Bohbot Y, Ringle A, Maréchaux S, Diouf M, Tribouilloy C (2018). Impact of low stroke volume on mortality in patients with severe aortic stenosis and preserved left ventricular ejection fraction. Eur Heart J.

[43] Eleid MF, Goel K, Murad MH, Erwin PJ, Suri RM, Greason KL, Nishimura RA, Rihal CS, Holmes DR (2015). Meta-Analysis of the prognostic impact of stroke volume, gradient, and ejection fraction after transcatheter aortic valve implantation. Am J Cardiol.

[44] Verma S, McMurray JJV (2018). SGLT2 inhibitors and mechanisms of cardiovascular benefit: a state-of-the-art review. Diabetologia.

[45] Chilton R, Tikkanen I, Cannon CP, Crowe S, Woerle HJ, Broedl UC, Johansen OE (2015). Effects of empagliflozin on blood pressure and markers of arterial stiffness and vascular resistance in patients with type 2 diabetes. Diabetes Obes Metab.

[46] Striepe K, Jumar A, Ott C, Karg MV, Schneider MP, Kannenkeril D, Schmieder RE (2017). Effects of the selective sodium-glucose cotransporter 2 Inhibitor empagliflozin on vascular function and central hemodynamics in patients with type 2 diabetes mellitus. Circulation.

[47] Solini A, Giannini L, Seghieri M, Vitolo E, Taddei S, Ghiadoni L, Bruno RM (2017). Dapagliflozin acutely improves endothelial dysfunction, reduces aortic stiffness and renal resistive index in type 2 diabetic patients: a pilot study. Cardiovasc Diabetol..

[48] Habibi J, Aroor AR, Sowers JR, Jia G, Hayden MR, Garro M, Barron B, Mayoux E, Rector RS, Whaley-Connell A, DeMarco VG (2017). Sodium glucose transporter 2 (SGLT2) inhibition with empagliflozin improves cardiac diastolic function in a female rodent model of diabetes. Cardiovasc Diabetol..

[49] Kidher E, Harling L, Ashrafian H, Naase H, Francis DP, Evans P, Athanasiou T (2014). Aortic stiffness as a marker of cardiac function and myocardial strain in patients undergoing aortic valve replacement. J Cardiothorac Surg..

[50] Obasare E, Bhalla V, Gajanana D, Rodriguez Ziccardi M, Codolosa JN, Figueredo VM, Morris DL, Pressman GS (2017). Natural history of severe aortic stenosis: diastolic wall strain as a novel prognostic marker. Echocardiography..

[51] Chirinos JA, Bhattacharya P, Kumar A, Proto E, Konda P, Segers P, Akers SR, Townsend RR, Zamani P (2019). Impact of diabetes mellitus on ventricular structure, arterial stiffness, and pulsatile hemodynamics in heart failure with preserved ejection fraction. J Am Heart Assoc..

